# Correlations of Gelling Properties and 3D Printability to the Chemical Composition and Rheological Properties of Surimi from Different Marine Fish Species

**DOI:** 10.3390/foods14030501

**Published:** 2025-02-05

**Authors:** Yijin Liu, Xinyan Tong, Jiajia Li, Ruizhi Yang, Zhengjie Liu, Xuezhi Shi, Bin Zheng, Fang Tian, Yadong Zhao

**Affiliations:** 1 School of Food and Pharmacy, Zhejiang Ocean University, Zhoushan 316022, China; 13797106241@163.com (Y.L.); 18867580748@163.com (X.T.); 15247473306@163.com (J.L.); ruizhiyang2001@163.com (R.Y.); liuzhengjie@zju.edu.cn (Z.L.); 6369958@163.com (B.Z.); 2 School of Marine Engineering Equipment, Zhejiang Ocean University, Zhoushan 316022, China; shixuezhi@zjou.edu.cn

**Keywords:** fish type, surimi, chemical composition, gel properties, 3D printing

## Abstract

Samples of marine surimi made from six different fish types have been comprehensively investigated and compared in terms of chemical composition, protease activity, gelling chemistry, gel properties and 3D printability. It has been found that surimi with high protein content, low fat concentration, low activity of endogenous protease (cathepsin B, cathepsin L, cathepsin D, calpain and serine protease) and low TVB-N showed better gelling capacity and gel properties. However, the 3D printability of surimi was more relevant to apparent viscosity. The surimi with viscosity between 5000 Pa·s and 12,000 Pa·s yielded better printing performance as indicated by higher printing accuracy than 97% and less cooking loss. This study clarified the effects of marine fish types on the corresponding surimi gelation and gel properties, and successfully established relationships between surimi properties and 3D printing performance, thus providing new insights on exploring new surimi sources and developing 3D surimi printing techniques.

## 1. Introduction

Surimi is an important aquatic product with broad market prospects. Surimi-based products are consumed worldwide due to their rich nutrients and texture attributes [1]. Until now, the major source of market-available surimi is marine fish, and this surimi is considered to have better gelling ability than that prepared from freshwater fish. For example, surimi made from both Gilthead Seabream (*Sqarus aurata*) and Meagre (*Argyrosomus regius*) had good gelling capacity. Their setting process included several stages of thermal gelation with higher transition temperature and larger storage modulus (G′) [2]. Surimi gels prepared from both Walleye Pollock (*Gadus chalcogrammus*) and White Croaker (*Pennahia argentata*) had great elasticity, and thus were regarded as good raw materials to prepare high-quality surimi-based products [3]. Hairtail (*Trichiurus lepturus*) is one of the major fish species that is used to prepare surimi in China. However, due to low salt-soluble protein content and high fat content, its surimi showed poor gelling ability and the resultant gel properties were unsatisfactory. It can be seen that different types of marine fish have been utilized to produce surimi and surimi products; however, the relationship of different fish species to surimi gel properties remains largely unknown.

The composition of surimi, being mainly protein, fat, and protease, affects the gelling process and the resultant gel quality. For example, myofibrillar proteins (MPs) were the main components responsible for gelation, and many properties of MPs would affect the properties of the gel, such as concentration, molecular weight and secondary structures [4]. In addition, the endogenous protease present in surimi could cause the degradation of MPs [5]. Then, the decomposition of myosin and actin could destroy the three-dimensional protein gel network [6], thus resulting in poor gel properties as indicated by an irreversibly reduced gel strength of surimi gels [7,8]. Fat was another important factor affecting surimi gel strength. It has been shown that the increase of fat content decreased the surimi gel strength, because the distance between adjacent MPs increased and the interaction or crosslinking between MPs were negatively influenced [9]. However, another study found that incorporating external fat could emulsify the surimi, thus improving the continuity and integrity of the surimi gel, and leading to better gel properties [7]. Although many available studies have been dedicated to investigating the effects of various surimi-related factors on the gel properties, the influence of the chemical composition of surimi made from different fish species on their gel properties has not been elucidated well.

At present, 3D printing technology is widely used in various fields, such as composite scaffolds [10], concrete buildings [11] and food [12]. Food 3D printing has been emerging as a modern additive manufacturing technique in the food industry [13]. It uses computer-aided layer-by-layer deposition to produce 3D food models. Due to its unique rheological properties and great gelling capacity, surimi has become a popular food ink. The choice of surimi ink is very important for 3D printing performance, although little attention has been paid to the exploration of different surimi sources for optimal surimi 3D printing performance.

After a literature search, we realized that no systematic study has been reported to investigate the relationship between the source of surimi and its 3D printing performance, which would hinder the exploitation of surimi as a food ink. To fill this knowledge gap, in this study, six kinds of surimi made from the most common marine fish in China or even worldwide [14,15], namely *Branchiostegus japonicus* (BJ), *Miichthys miiuy* (MM), *Pagrosomus major* (PM), *Ilisha elongate* (IE), *Aspitrigla cuculus* (AC) and *Trichiurus lepturus* (TL), have been comprehensively investigated and compared in terms of chemical composition and protease activity. Furthermore, their relationships to surimi gel properties and 3D printing performance have been well established. It is expected that the results obtained in this study will serve as a foundation for the selection of suitable raw materials for specific surimi processing, such as food 3D printing.

## 2. Materials and Methods

### 2.1. Materials

Frozen *Branchiostegus japonicus* surimi (grade AAA), frozen *Miichthys miiuy* surimi (grade AAA), frozen *Pagrosomus major* surimi (grade AAA), frozen *Ilisha elongate* surimi (grade AAA), frozen *Aspitrigla cuculus* surimi (grade AAA), and frozen *Trichiurus lepturus* surimi (grade AAA) were purchased from Zhejiang Xingye Group Co., Ltd. (Zhoushan, China). PVC plastic transparent casings were bought from Tianjin Kangtai Plastic Packaging Co., Ltd. (Tianjin, China). Edible salt was purchased from local shops.

### 2.2. Overview of Experimental Design

**Figure 1:**
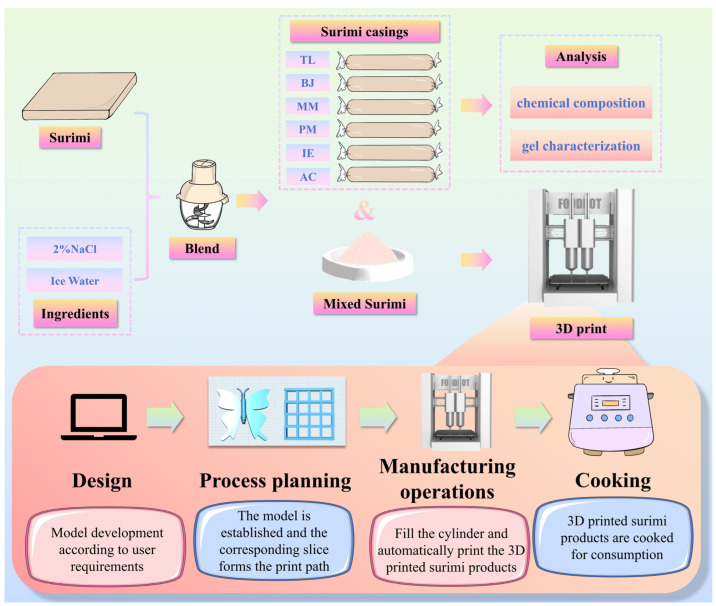
Workflow diagram of the experimental design in this study.

In order to investigate the correlations of gelling properties and 3D printability to surimi produced from different marine fish species, the experimental design shown in Figure 1 was followed. First, the chemical composition and rheological properties of six kinds of surimi were analyzed, and then the 3D printability and the printing performance of the surimi were investigated. After that, the 3D-printed structures were subjected to heating, and the gel characteristics of the obtained surimi gels were studied. Upon completing these experiments, the relationship of the surimi’s source to its 3D printability and gel properties would be established.

### 2.3. Preparation of Surimi Gels

Frozen surimi was thawed at 4 °C overnight. Each surimi sample (300 g) was blended for 1 min, and then 2% NaCl was added for another cycle of blending for 2 min. Ice water was added to adjust the water content to 78%, and mixed under 10 °C for 2 min. The obtained surimi paste was placed into transparent PVC plastic casings with a diameter of 25 mm. The casings filled with surimi were first heated at 40 °C for 30 min and then at 90 °C for 20 min. After cooling down in an ice bath for 30 min, the prepared surimi gels were stored at 4 °C overnight until further analysis.

### 2.4. Surimi Chemical Composition Analysis

#### 2.4.1. Protein

The protein content in the surimi was determined according to GB5009.5-2016 [16]. A 1 g surimi sample was placed in the digestive tube, then sulfuric acid (20 mL), potassium sulfate (6 g) and copper sulfate (0.4 g) were added. The mixture was digested in a furnace at 420 °C for 1 h to obtain a transparent green liquid. Then, 50 mL water was added, and the sample was subjected to distillation, titration and analysis by employing an automatic Kjeldahl nitrogen analyzer.

#### 2.4.2. Fat

The fat content in the surimi was tested according to GB5009.6-2016 [17]. We weighed a 3 g surimi sample and put it into a filter paper tube. We then put the filter cartridge into the Soxhlet extractor, and heated the water bath to make the petroleum ether continuously reflux for fat extraction; the extraction time was 8 h. The petroleum ether was recovered, and then evaporated in the water bath and dried at 100 °C for 1 h. After cooling in the dryer for 0.5 h, the weight was recorded until the difference between the two-weighing data was no more than 2 mg.

#### 2.4.3. Ash

The ash in the surimi was tested according to GB5009.4-2016 [18]. A 3 g sample was weighed and 1 mL of 24% (*w*/*v*) magnesium acetate solution was added. The mixture stood by for 10 min until a water bath was used to evaporate the water. After evaporation, the sample was fully carbonized by small fire heating, and then placed in a high-temperature furnace and burned at 550 °C for 4 h. After cooling to 200 °C, the samples were placed in a dryer to cool for 30 min and then weighed. Repeated burning was performed until the difference between the two-weighing data did not exceed 0.5 mg. The ash content ash was calculated based on the obtained $m_{0}$ (weight of magnesium oxide), $m_{1}$ (weight of crucible and ash), $m_{2}$ (weight of crucible), and $m_{3}$ (weight of crucible and sample) values using the following formula:

$$Ash\;content\;(\%)=\frac{m_{1}-m_{2}-m_{0}}{m_{3}-m_{2}}\times 100$$

#### 2.4.4. Total Volatile Basic Nitrogen (TVB-N)

The TVB-N of the surimi sample was determined according to the semi-micro titration method (GB 5009.228-2016 [19]). First, 10 mL supernatant liquid from the surimi sample and 5 mL magnesium oxide suspension at 1% (*w*/*v*) were added to the Kjeldahl apparatus (KDN-818, Ruifeng Experimental Equipment Co., Ltd., Guangzhou, China) and then subjected to distillation for 5 min. The obtained distillate was mixed with 10 mL aqueous boric acid at 2% (*w*/*v*) containing five droplets of indicator. Hydrochloric acid (HCl) solution at 0.01 M was used to titrate the solution and the TVB-N was calculated.

#### 2.4.5. Protease Activity

A 2 g surimi sample was placed in a centrifuge tube, and 18 mL PBS solution was added before homogenization at 12,000 r for 1 min. The sample was centrifuged at 8000 r/min for 15 min, the supernatant was taken, and the protease activity of the surimi was determined with an ELISA assay kit bought from Beijing Solarbio Science & Technology Co., Ltd. (Beijing, China).

### 2.5. Surimi Gel Characterization

#### 2.5.1. Whiteness

The surimi gel was cut into cubes with a side length of 1cm and then analyzed with a colorimeter (Shenzhen Sanen Chi Technology Co., Ltd., Shenzhen, China). The color parameters L*, a* and b* of the surimi gel represented the lightness, red-greenness and blue-yellowness, respectively. The following formula recommended by the National Fisheries Research Institute was used to calculate whiteness:

$$Whiteness=100-{\left[{\left(100-L^{*}\right)}^{2}+a^{*2}+b^{*2}\right]}^{\frac{1}{2}}$$

#### 2.5.2. Water Holding Capacity

A 3 g sample of surimi gel was weighed as $m_{1}$. The surimi gel was covered with two layers of filter paper, and was then loaded into a 50 mL centrifuge tube and then centrifuged at 8000 r/min for 10 min. After this treatment, the surimi gel was weighed as $m_{2}$, and the WHC could be calculated according to the following formula:

$$WHC\left(\%\right)=\frac{m_{1}}{m_{2}}\times 100$$

#### 2.5.3. Texture Profile Analysis (TPA)

Surimi gel in a cylinder shape of 25 mm × 25 mm was analyzed with a texture analyzer (Model TA-XT2, Stable MicroSystems, Surrey, UK). A cylindrical P/36R probe with a diameter of 36 mm was used. The compression test was performed at a compression speed of 3 mm/s and a compression distance of 5 mm/s. Each sample was measured seven times to obtain the average values.

#### 2.5.4. Dynamic Rheological Test

An AR 2000 ex dynamic rheometer (TA Instrument Ltd., New Castle, DE, USA) was used to analyze the rheological properties of surimi gels. The surimi paste was placed between two parallel holders, and the interval was set at 1 mm. After relaxing for 2 min, the samples were heated at 4 °C/min from 20 °C to 90 °C. The storage modulus (G′) and loss modulus (G″) were measured when the stress of 10 Pa and the frequency of 1 Hz were applied. All samples were measured three times.

#### 2.5.5. Scanning Electron Microscopy (SEM)

Surimi gel cylinders with a thickness of 0.5 mm were dried in a freeze dryer for 48 h. The dried surimi gel was coated with gold at a thickness of 3–5 nm using a Cressington208 HR high-resolution sputtering instrument. A Hitachi S-4800 field emission scanning electron microscope (SEM) (Guoyi Quantum Technology Co., Ltd., Hefei, China) was employed to observe the morphology of the samples.

#### 2.5.6. SDS-PAGE

A 2 g sample of surimi gel was extracted by SDS (5 g/100 mL) at 85 °C for 1 h and then 95 °C for 10 min. It was then centrifuged at 10,000 r/min for 30 min to obtain the supernatant. A BCA kit was used to determine the protein concentration, and the concentration was set to 7 mg/100 g. The supernatant was mixed with a protein buffer (5×) at a ratio of 1:3, placed in a boiling water bath for 2 min, and centrifuged at 12,000 r/min for 2 min. US = 80 V, IS = 20 Ma and TS = 6 min were used for gel analysis. Finally, the gel was taken out and Coomassie brilliant blue R-250 staining solution was added, after which the gel was left to stand still for 2 h. At the end of the staining, the decolorization solution (glacial acetic acid/water/ethanol = 1:5:4) was added, and the gel was kept still for about 2 h. Repeated decolorization was conducted until the gel had no background color.

### 2.6. Evaluation of 3D Printing Performance

#### 2.6.1. Preparation of Surimi Ink

Frozen surimi (200 g) was thawed at 4 °C for 12 h. Then, 2% NaCl was added and the mixture was blended with a blender (Shenzhen Bodi Culture Media Co., Ltd., Shenzhen, China) to prepare the inks for 3D printing.

#### 2.6.2. Viscosity Determination

Dynamic oscillation frequency within 0.1 to 100 Hz was performed on an AR 2000 ex dynamic rheometer (TA Instrument Ltd., New Castle, DE, USA) to analyze the viscosity of the surimi inks.

#### 2.6.3. 3D Printing

A FOODBOT-MAT extrusion 3D printer (SHIYIN Technologies Co., Ltd., Hangzhou, China) was used for a 3D printing test. The 123D Design software (2.2.14) was used to design two models, a grid (50 × 50 × 10 mm) and a butterfly (45 × 50 × 5 mm). A nozzle diameter of 0.84 mm, a layer height of 0.5 mm and a printing speed of 70 mm/s were used for printing at room temperature (25 °C).

#### 2.6.4. Printing Accuracy Analysis

The printing accuracy was analyzed by referring to the method of Cheng et al. [20]. After printing the grid, the three dimensions (length, width and height) of the 3D printed models were measured with a vernier caliper. The 3D printing accuracy was calculated with the following formula:

$$Printing\;accuracy\left(\%\right)=(1-\frac{\left|D-D_{A}\right|}{D_{A}})\times 100$$

where D was the measured value, mm; D_A_ was the predesigned model value, mm.

#### 2.6.5. Cooking Loss

The weight changes of the 3D printed samples before and after cooking were used to calculate the cooking loss (%) with the formula below:

$$Cooking\;loss\left(\%\right)=\frac{m_{1}-m_{2}}{m_{1}}\times 100$$

where m_1_ was the weight of the 3D printed samples before heating (g) and m_2_ was the weight of the 3D printed samples after heating (g).

### 2.7. Statistical Analysis

Each test was performed at least three times to obtain the mean values. The significant differences (*p* < 0.05) were determined based on Analysis of variance (ANOVA) and Duncan’s multiple ranges using SPSS 27 software [10].

## 3. Results and Discussion

### 3.1. Chemical Composition of Surimi from Different Fish

**Figure 2:**
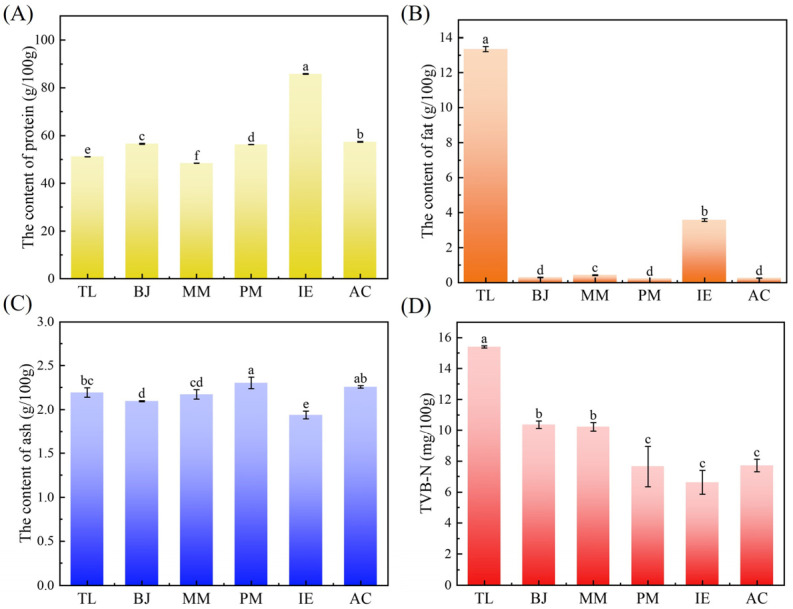
Chemical components of surimi samples made from different fish. (**A**) Protein, (**B**) fat, (**C**) ash and (**D**) TVB-N. Different lowercase letters ^(a–f)^ in the same group indicate statistically significant differences at *p* < 0.05.

The main component of surimi is myofibrillar protein, which plays a crucial role in maintaining the texture and physicochemical properties of surimi gel [21]. As shown in Figure 2A, the protein contents in surimi made from six different fish species were generally high (>45 g/100 g). The protein content of IE (86 g/100 g) was significantly higher than those of TL, MM, PM, BJ and AC (*p* < 0.05). The protein content of TL (51.1 g/100 g) was lowest. The difference in surimi protein content could be due either to the high protein characteristics of the fish species itself or to the rinsing processing during surimi preparation.

Various surimi from different fish species also showed diversities in fat content. As shown in Figure 2B, the fat content of TL surimi (13.5 g/100 g) was significantly higher than those of other surimi samples. It was followed by IE (3.5 g/100 g). The fat contents of BJ, MM, PM and AC were relatively low, lower than 0.4 g/100 g. During rinsing of the surimi, large amounts of lipid components were removed while concentrated myofibrillar proteins were retained to improve the storage stability of the surimi and the gel quality of the surimi products [22], meaning that the fat contents of the surimi samples were quite low. According to previous findings, TL surimi had quite a high fat content and contained a large amount of unsaturated fatty acids compared with other types of surimi [23]. During gelation of the surimi, the fat oxidation seemed to cause degradation of myofibrillar proteins, thus leading to tissue softening. This indicated that fat must be an important factor affecting surimi gelation and gel properties.

Total ash refers to the remaining inorganic matter after high-temperature combustion, which can reflect the mineral content. As shown in Figure 2C, the ash contents of the six surimi samples were slightly different, ranging from 1.94% to 2.26%, of which the ash content of IE was lowest.

TVB-N was usually used as an indicator to evaluate the freshness of high-protein foods, and was also able to reflect the quality of the surimi [24]. During surimi preparation and storage, bacteria and enzymes could decompose proteins, thus releasing ammonia and other nitrogen-containing compounds to bind TVB-N with other substances in the surimi. Therefore, the higher the TVB-N value, the lower the freshness of the surimi. The TVB-N value of TL was 15.4 mg/100 g (Figure 2D), which was significantly higher than that of other groups (*p* < 0.05). The TVB-N values were 10.36 mg/100 g, 10.22 mg/100 g, 7.89 mg/100 g and 7.37 mg/100 g for BJ, MM, PM and AC, respectively (Figure 2D). The lowest TVB-N value of IE was 6.53 mg/100 g, suggesting that it was the freshest.

### 3.2. Protease Activities of Surimi Samples Made from Different Fish

**Figure 3:**
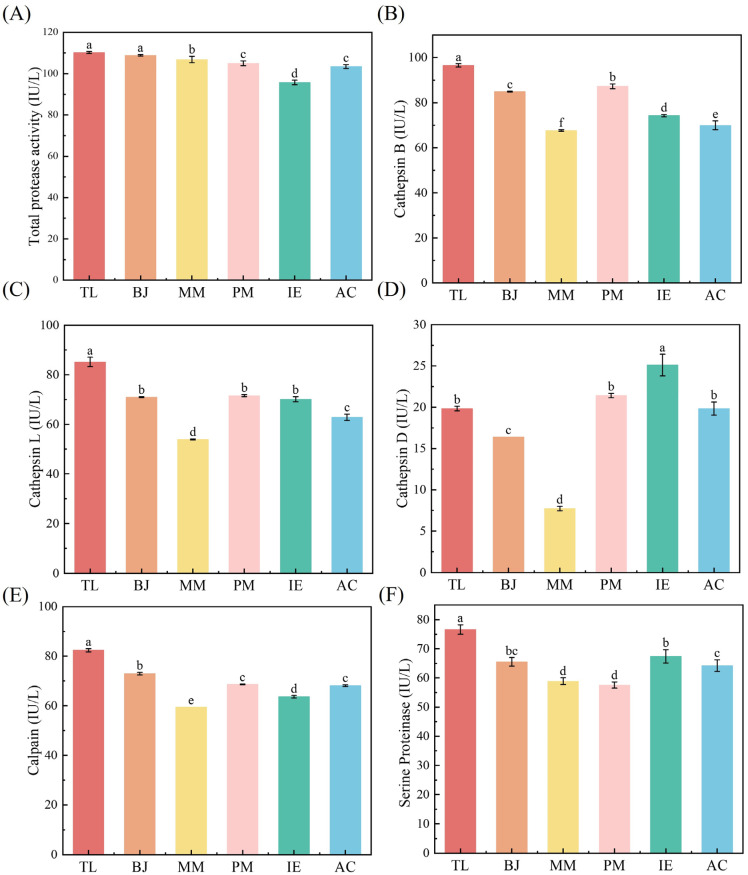
Protease activities of surimi samples made from different fish. (**A**) Total protease activity, (**B**) cathepsin B, (**C**) cathepsin L, (**D**) cathepsin D, (**E**) calpain and (**F**) serine protease. Different lowercase letters ^(a–f)^ in the same group indicate statistically significant differences at *p* < 0.05.

Endogenous protease in surimi could be activated under the heating conditions at 50~70 °C, which accelerated the degradation rate of myofibrillar proteins and led to a decrease in gel strength, resulting in irreversible damage to the gel structure of surimi gels [25]. As shown in Figure 3A, TL showed the highest total protease activity (110.22 IU/L), while the lowest value was found for IE (95.76 IU/L).

Myosin and actin are two important proteins that affect the gelation ability and gel quality of surimi. However, cysteine protease could cause the degradation of myosin and actin during thermal processing, thus resulting in surimi gel deterioration [6]. Cathepsin B and L are the two main cysteine proteases. The activity of cathepsin B and cathepsin L in TL surimi were highest among all surimi samples, 96.57 IU/L and 85.17 IU/L, respectively, while MM showed the lowest activities of cathepsin B and cathepsin L at 67.64 IU/L and 53.92 IU/L, separately (Figure 3B,C).

Cathepsin D was also a contributor to surimi gel deterioration, and could hydrolyze the myosin heavy chain (MHC), myosin light chain, actin, co-actin and tropomyosin, but could not degrade myosin [26]. The cathepsin D activities of TL, BJ, MM, PM, IE and AC surimi samples were 19.84 IU/L, 16.42 IU/L, 7.74 IU/L, 21.42 IU/L, 25.11 IU/L and 19.84 IU/L, respectively (Figure 3D). It can be seen that the cathepsin D activities were relatively low compared to previously mentioned cathepsin B and L. This might be due either to its removal during rinsing in surimi preparation or to inactivation/denaturation at a high temperature [27].

Calpain is a calcium-dependent intracellular neutral cysteine protease. Like cathepsin, calpain had a great influence on the degradation of myofibrillar protein, thus affecting the gel properties of surimi [28]. Calpain was mainly distributed near the Z line (or Z disk) at the sarcomere junction of skeletal muscle, and acted on the Z line to decompose and destroy myofibrillar protein [29]. As shown in Figure 3E, calpain induced different active reactions in different kinds of surimi. Among them, the calpain activity of TL was the highest (82.43 IU/L), followed by BJ at 72.93 IU/L, and the lowest at 59.43 IU/L for MM. Serine protease was able to degrade the myosin heavy chain (MHC), actin and tropomyosin of surimi under heating at 60 °C. The activity of serine protease in TL was 76.63 IU/L, while values of about 60 IU/L were found for the other five surimi samples.

### 3.3. Gel Properties of Surimi Gels Made from Different Fish

#### 3.3.1. Dynamic Rheological Characteristics

**Figure 4:**
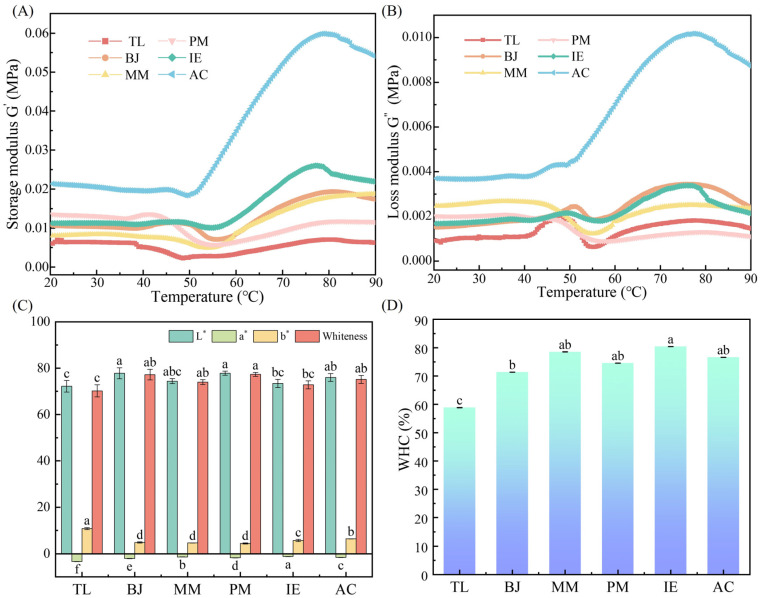
Dynamic rheological properties, color parameters and WHC of surimi gels made from different fish. (**A**) Storage modulus G′, (**B**) loss modulus G″, (**C**) color parameters and (**D**) WHC. Different lowercase letters ^(a–c)^ in the same group indicate statistically significant differences at *p* < 0.05.

The dynamic rheological properties of six different surimi samples were analyzed upon heating to visualize the gelation process. The storage modulus (G′) represented the energy stored in the gel during the shear process, which was related to the elasticity [30] (Figure 4A); the loss modulus (G″) represented the dissipation and loss of energy in the gel during the shear process, which was related to viscosity (Figure 4B). As the temperature increased, generally the G′ of all the surimi gels first stood stable and then increased rapidly to the maximum, followed by a slight decrease (Figure 4A). Among all of the samples, AC showed the better gelation profiles due to its higher protein content, lower fat content and protease activities. For AC, G′ was stable until 50 °C, and then increased from 50 °C to 75 °C due to the formation of initial gel at the myosin denaturation temperature, resulting in a partial unfolding of myosin. Subsequently, G′ slightly decreased from 75 °C to 90 °C, indicating the non-covalent bond cleavage in surimi, which might be due to the decomposition of myosin or protein molecular rearrangement by endogenous protease [31]. Compared with AC, all of the other surimi samples showed much poorer gelling capacities, with the worst case being TL, for which no obvious gelation phenomenon was observed and its G′ was always lower than all of the others. This might be due to its high endogenous protease activity and high fat content.

#### 3.3.2. Protein Patterns

**Figure 5:**
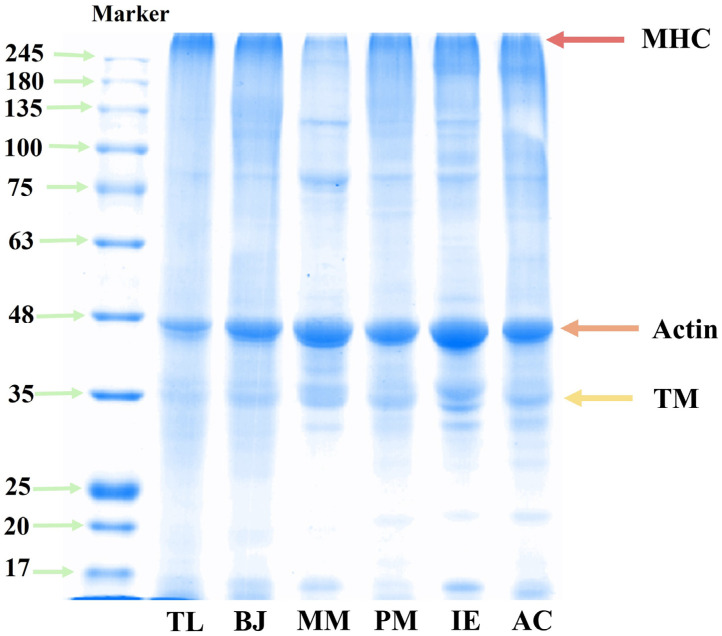
SDS-PAGE analysis of surimi gels made from different fish.

SDS-PAGE was used to analyze the differences in protein profiles of different kinds of surimi gel samples (Figure 5). The typical bands of surimi proteins were clearly visible, including myosin heavy chain (MHC, 220 kDa), actin (Actin, 48 kDa) and troponin (TM, 35 kDa) [11]. MHC and actin bands were significantly more obvious than other bands, and they were the two most important proteins for gel formation [32]. The MHC bands were almost invisible for all surimi gels, indicating that these large molecular weight proteins were degraded due to the hydrolytic effects of endogenous protease during the heating process. In MM, the contents of actin and TM were higher than other samples as indicated by higher intensities of bands at 48 kDa and 35 kDa, thus being beneficial to gel formation. The bands of TL were significantly lighter than those of other surimi gels, agreeing well with its having the lowest protein content. In addition, the higher endogenous protease activity of TL would cause the degradation of high molecular weight proteins into low molecular weight proteins or peptides, meaning that the intensities for the bands of MHC, actin and TM were reduced.

#### 3.3.3. Color Parameters

The color characteristics of surimi gel mainly depended on the properties of surimi, and they were important indexes to evaluate the quality of surimi products. In general, consumers preferred surimi products with high L* values [33]. It can be seen that the six kinds of surimi gel showed different whiteness values, among which the highest value of 77.31 was found for PM (Figure 3C). The whiteness value (70.2) and L* value (72.17) of TL surimi gel were the lowest, but its b* (yellowness value, 10.7) was significantly higher than those of other surimi gels (*p* < 0.05). This might be due to the high fat content of TL which could be oxidized to generate colorful components. The difference in the L*, a*, b* and whiteness values of different surimi gels should be related to the chemical composition of different fish species. When the hemoglobin content in fish muscle tissue was high, the a* value of surimi gel would be large, which could then reduce the brightness L* and whiteness [34]. In addition, the content of metmyoglobin, oxymyoglobin and deoxymyoglobin in different fish species and their sensitivity to the environment were different, resulting in differences in whiteness [35,36].

#### 3.3.4. Network and Microstructures

**Figure 6:**
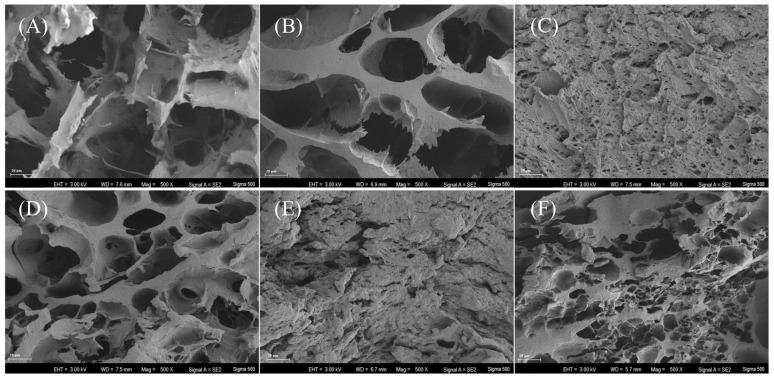
SEM images at ×300 magnification of surimi gels made from different fish. (**A**) TL, (**B**) BJ, (**C**) MM, (**D**) PM, (**E**) IE and (**F**) AC.

The microscopic observation of surimi gels reflected the quality of the three-dimensional network structure formed by the cross-linking of myosin and actin. In all surimi gels, certain cavities could be observed, which may have been caused by the diffusion and expansion of water, fat particles and air [37]. Among them, the network structures of MM and IE were much finer and more compact, and the pores were smaller than those of other surimi gels, indicating their better gel quality (Figure 6). Although AC showed a quite ordered and dense network, the pore sizes were much larger than MM and IE. Very porous structures with big voids were observed for BJ, PM and TL. In particular, the gel network of TL was characterized by the largest pores and loosest structure, suggesting the poorest gel profiles. TL showed the highest endogenous protease activity, thus inducing a serious degradation of surimi proteins and leading to the obvious collapse of the surimi gel network [38].

#### 3.3.5. Water Holding Capacity

WHC was an important index of surimi gel quality. It reflected the fluidity of free water in the three-dimensional network of surimi gel, and indirectly indicated the integrity and stability of the surimi gels’ structure [39]. The difference in WHC of the six surimi gels is shown in Figure 3D. The WHC of the BJ, MM, PM, IE and AC surimi gels was maintained at about 80%, while the lowest WHC was observed for TL surimi gel (58.85%). It should be noted that WHC was related to the microstructure of the surimi gels. For example, the gel network of TL had large voids, a loose structure and low gel density, which may have increased the fluidity of free water and led to low WHC. For the surimi gels with compact gel network structures and small voids, the WHC was generally high. In addition, the WHC of surimi gel also had a certain correlation with the freshness of surimi as indicated by the TVB-N values. In fact, the structure of myofibrillar proteins in fresh surimi was relatively complete, meaning that it could entrap water more effectively.

#### 3.3.6. Texture Properties

Texture property analysis (TPA) was able to accurately reveal the gel properties of surimi gels. During the TPA test, the sample was squeezed twice to simulate chewing behaviors [40]. There were some differences in hardness, gumminess and chewiness, and the difference in chewiness was the most significant (*p* < 0.05) (Table 1). Chewiness was the degree of difficulty in chewing the surimi gels, which were between 323 and 2305 for all of the surimi gels. Among them, AC showed the highest hardness, gumminess and chewiness, suggesting that its gel characteristics were the best. This was followed by IE, PM, MM and BJ, in that order. The lowest values were found for TL, 534, 361 and 323 for hardness, gumminess and chewiness, respectively. This confirmed that TL had the poorest gel quality due to low protein, high fat, great protease activity and large TVB-N.

### 3.4. 3D Printing Performance of Surimi Inks Made from Different Fish

**Figure 7:**
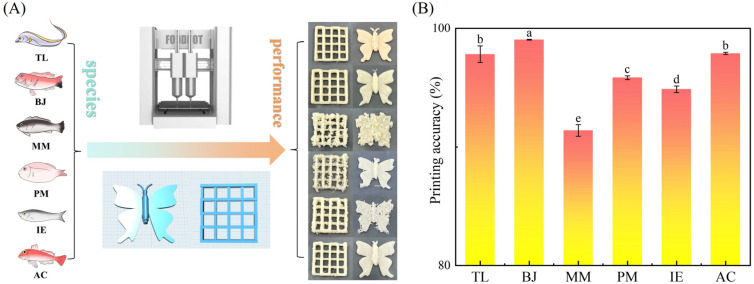
Printability and printing fidelity evaluation of surimi inks made from different fish. (**A**) Appearance of 3D printed surimi structures and (**B**) printing accuracy. Different lowercase letters ^(a–e)^ in the same group indicate statistically significant differences at *p* < 0.05.

In order to evaluate the printability and printing fidelity of surimi inks made from different fish species, two different 3D print models, grid and butterfly, were designed and utilized. It can be seen that the printed structures of TL, BJ and AC showed better printability and higher printing fidelity, but the performances of IE, MM and PM were much poorer as indicated the irregular shapes (Figure 7A). This was also supported by the quantitative analysis of the printing accuracy. As shown in Figure 7B, BJ had the highest printing accuracy of 99.04%, followed by AC and TL at around 98%. The printing accuracy values were 95.84% and 94.87% for PM and IE, respectively. MM showed the lowest printing accuracy of 91.39%, suggesting that it was almost non-printable by the 3D printer.

**Figure 8:**
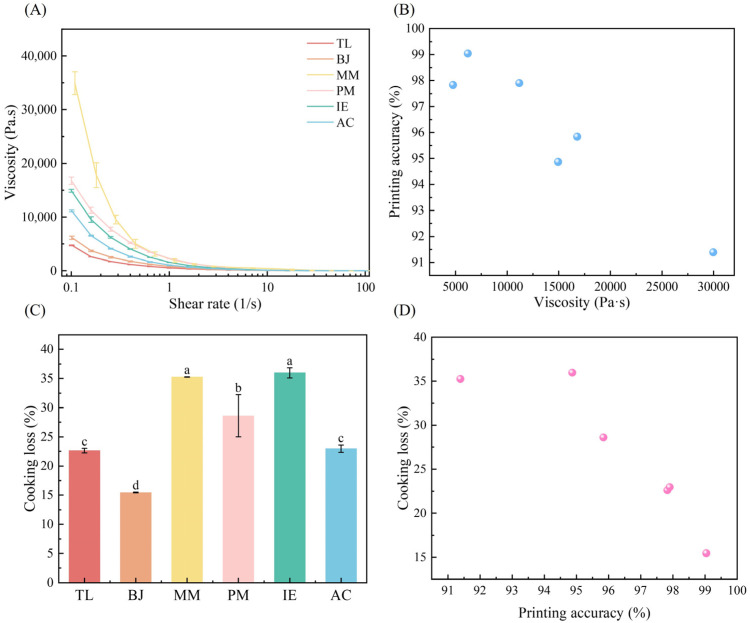
Correlation between surimi ink property and 3D printing performance. (**A**) viscosity of surimi inks, (**B**) correlation between viscosity and printing accuracy, (**C**) cooking loss, (**D**) correlation between printing accuracy and cooking loss. Different lowercase letters ^(a–d)^ in the same group indicate statistically significant differences at *p* < 0.05.

Viscosity changes of different surimi samples against the increasing shear rate (from 0.1 to 100 s^−1^) were analyzed and shown in Figure 8A. It can be seen that all of the surimi samples were pseudoplastic fluids with shear thinning properties [16]. MM had the highest viscosity, followed by IE, PM, AC and BJ, while TL had the lowest viscosity. It should be noted that a certain correlation between the shape stability of the 3D printed structures and the viscosity of surimi inks has been found, as shown in Figure 8B, namely that the lower the viscosity, the higher the printing accuracy. Highly viscous surimi inks, such as MM, IE and PM, were more difficult to extrude from the printing tip than other surimi inks with lower viscosity, meaning that the 3D printed surimi products showed poor structures. According to the results obtained in this study, the surimi inks with viscosities ranging from 5000 Pa·s to 12,000 Pa·s could yield great printing performances with 3D printing accuracy higher than 97%.

When the 3D printed models were subjected to cooking, the quality of the obtained 3D printed surimi gel products was assessed in terms of cooking loss. As shown in Figure 8C, IE, MM and PM had higher cooking loss than AC, TL and BJ. It was interesting to see a certain correlation between cooking loss and printing accuracy (Figure 8D); the higher the printing accuracy, the lower the cooking loss. The 3D printed structures of IE, MM and PM were randomly arranged with rough surfaces and porous networks, so that the soluble ingredients in the surimi gels could be easily leached and the cooking loss could increase.

## 4. Conclusions

The chemical composition of six marine fish types, *Branchiostegus japonicus* (BJ), *Miichthys miiuy* (MM), *Pagrosomus major* (PM), *Ilisha elongate* (IE), *Aspitrigla cuculus* (AC) and *Trichiurus lepturus* (TL), have been analyzed, and were further correlated to their different gel properties and 3D printing performance. IE had the highest protein content, lowest protease activity and lowest TVB-N, thus contributing to its having the best gel profiles. The fat content of TL was significantly higher than surimi samples made from other fish species, resulting in serious gel deterioration. Surimi samples made from MM, IE and PM with higher viscosity values could not be extruded well, meaning that poorer 3D printability and lower printing fidelity were observed. On the contrary, surimi made from BJ, AC and TL with viscosity between 5000 Pa·s and 12,000 Pa·s resulted in greater shape stability and less cooking loss. The results obtained in this study could serve as a basis for exploring new surimi sources and introducing modern 3D printing techniques into the traditional surimi industry.

## Figures and Tables

**Table 1:** TPA attributes of surimi gels made from different fish *.

Group	Hardness (g)	Springiness	Cohesiveness	Gumminess	Chewiness (g)	Resilience
TL	534 ± 77 ^e^	0.85 ± 0.05 ^c^	0.63 ± 0.05 ^e^	361 ± 41 ^d^	323 ± 15 ^d^	1.49 ± 0.21 ^f^
BJ	1202 ± 112 ^d^	0.93 ± 0.00 ^b^	0.70 ± 0.01 ^d^	1010 ± 34 ^c^	942 ± 16 ^c^	1.73 ± 0.03 ^e^
MM	1470 ± 205 ^cd^	0.98 ± 0.00 ^a^	0.82 ± 0.00 ^a^	1269 ± 140 ^bc^	1246 ± 133 ^bc^	2.57 ± 0.02 ^a^
PM	1795 ± 102 ^c^	0.96 ± 0.02 ^ab^	0.75 ± 0.01 ^bc^	1428 ± 137 ^b^	1359 ± 120 ^b^	2.11 ± 0.07 ^c^
IE	2810 ± 181 ^b^	0.99 ± 0.01 ^a^	0.78 ± 0.00 ^b^	2217 ± 272 ^a^	2185 ± 322 ^a^	2.32 ± 0.02 ^b^
AC	3362 ± 348 ^a^	0.93 ± 0.00 ^b^	0.74 ± 0.00 ^c^	2473 ± 202 ^a^	2305 ± 258 ^a^	1.92 ± 0.03 ^d^

* Values are presented as mean ± SD. Different superscripts in the same column indicate significant differences at *p* < 0.05.

## Data Availability

The original contributions presented in the study are included in the article/Supplementary Materials, further inquiries can be directed to the corresponding authors.

## Supplementary Materials

The following supporting information can be downloaded at: https://www.mdpi.com/article/10.3390/foods14030501/s1, Supplementary Materials S1. One-way ANOVA of the materials. Supplementary Materials S2. Calculated errors of the chemical composition.
