# Supplementary material for: Correlations of Gelling Properties and 3D Printability to the Chemical Composition and Rheological Properties of Surimi from Different Marine Fish Species
Source: Foods. 2025 Feb 5;14(3):501. doi: 10.3390/foods14030501 (PMC11816489; doi:10.3390/foods14030501)
Supplement: Supplementary file 1 [file foods-14-00501-s001.zip › foods-3421327-supplementary.pdf]

Supplementary Materials S1. One-way ANOVA of the materials

Table S1 Results of One-way ANOVA comparison of protein between different surimi

Source of variation	DF	SS	MS	F	*P* value
Protein (g/100g)	5	2720.590	544.118	21764.720	0.000
Error	12	0.300	0.025		
Total	17	2720.890			

Table S2 Results of One-way ANOVA comparison of fat between different surimi

Source of variation	DF	SS	MS	F	*P* value
Fat (g/100g)	5	410.245	82.049	16687.926	0.000
Error	12	0.059	0.005		
Total	17	410.304			

Table S3 Results of One-way ANOVA comparison of ash between different surimi

Source of variation	DF	SS	MS	F	*P* value
Ash (g/100g)	5	0.249	0.050	24.637	0.000
Error	12	0.024	0.002		
Total	17	0.273			

Table S4 Results of One-way ANOVA comparison of TVB-N between different surimi

Source of variation	DF	SS	MS	F	*P* value

TVB-N (g/100g)	5	152.262	30.452	59.571	0.000
Error	12	6.134	0.511		
Total	17	158.397			

Table S5 Results of One-way ANOVA comparison of total protease activity between different surimi

Source of variation	DF	SS	MS	F	*P* value
Total protease activity (IU/L)	5	400.896	80.179	80.124	0.000
Error	12	12.008	1.001		
Total	17	412.904			

Table S6 Results of One-way ANOVA comparison of cathepsin B between different surimi

Source of variation	DF	SS	MS	F	*P* value
Cathepsin B (IU/L)	5	1916.215	383.243	385.620	0.000
Error	12	11.926	0.994		
Total	17	1928.141			

Table S7 Results of One-way ANOVA comparison of cathepsin L between different surimi

Source of variation	DF	SS	MS	F	*P* value
Cathepsin L (IU/L)	5	1615.625	323.125	301.816	0.000
Error	12	12.847	1.071		

Total	17	1628.472			

Table S8 Results of One-way ANOVA comparison of cathepsin D between different surimi

Source of variation	DF	SS	MS	F	*P* value
Cathepsin D (IU/L)	5	527.597	105.519	247.086	0.000
Error	12	5.125	0.427		
Total	17	532.722			

Table S9 Results of One-way ANOVA comparison of calpain between different surimi

Source of variation	DF	SS	MS	F	*P* value
Calpain (IU/L)	5	952.125	190.425	1054.662	0.000
Error	12	2.167	0.181		
Total	17	954.292			

Table S10 Results of One-way ANOVA comparison of serine protease between different surimi

Source of variation	DF	SS	MS	F	*P* value
Serine protease (IU/L)	5	703.831	140.766	51.584	0.000
Error	12	32.747	2.729		
Total	17	736.578			

Table S11 Results of One-way ANOVA comparison of L* between different surimi gels

Source of variation	DF	SS	MS	F	*P* value
L*	5	81.910	16.382	4.960	0.011
Error	12	39.633	3.303		
Total	17	121.543			

Table S12 Results of One-way ANOVA comparison of a* between different surimi gels

Source of variation	DF	SS	MS	F	*P* value
a*	5	9.180	1.836	435.427	0.000
Error	12	0.051	0.004		
Total	17	9.231			

Table S13 Results of One-way ANOVA comparison of b* between different surimi gels

Source of variation	DF	SS	MS	F	*P* value
b*	5	85.949	17.190	159.905	0.000
Error	12	1.290	0.107		
Total	17	87.239			

Table S14 Results of One-way ANOVA comparison of whiteness between different surimi gels

Source of variation	DF	SS	MS	F	*P* value
whiteness*	5	111.563	22.313	6.712	0.003

Error	12	39.891	3.324		
Total	17	151.454			

Table S15 Results of One-way ANOVA comparison of WHC between different surimi gels

Source of variation	DF	SS	MS	F	*P* value
WHC (%)	5	910.375	182.075	12.794	0.000
Error	12	170.776	14.231		
Total	17	1081.152			

Table S16 Results of One-way ANOVA comparison of hardness between different surimi gels

Source of variation	DF	SS	MS	F	*P* value
Hardness (g)	5	16517998.19	3303599.638	88.048	0.000
Error	12	450245.600	37520.467		
Total	17	16968243.79			

Table S17 Results of One-way ANOVA comparison of springiness between different surimi gels

Source of variation	DF	SS	MS	F	*P* value
Springiness	5	0.040	0.008	16.234	0.000
Error	12	0.006	0.000		
Total	17	0.046			

Table S18 Results of One-way ANOVA comparison of ccohesiveness between different surimi gels

Source of variation	DF	SS	MS	F	*P* value
Cohesiveness	5	0.063	0.013	32.966	0.000
Error	12	0.005	0.000		
Total	17	0.068			

Table S19 Results of One-way ANOVA comparison of ggumminess between different surimi gels

Source of variation	DF	SS	MS	F	*P* value
Gumminess	5	9139651.967	1827930.393	70.374	0.000
Error	12	311694.843	25974.570		
Total	17	9451346.811			

Table S20 Results of One-way ANOVA comparison of chewiness between different surimi gels

Source of variation	DF	SS	MS	F	*P* value
Chewiness (g)	5	8492880.467	1698576.093	50.173	0.000
Error	12	406256.029	33854.669		
Total	17	88899136.496			

Table S21 Results of One-way ANOVA comparison of resilience between different surimi gels

Source of variation	DF	SS	MS	F	*P* value
Resilience	5	2.328	0.466	54.932	0.000
Error	12	0.102	0.008		
Total	17	2.430			

Table S22 Results of One-way ANOVA comparison of printing accuracy between different surimi

Source of variation	DF	SS	MS	F	*P* value
Printing accuracy (%)	5	115.956	23.191	166.856	0.000
Error	12	1.668	0.139		
Total	17	78.995			

Table S23 Results of One-way ANOVA comparison of cooking loss between different surimi

Source of variation	DF	SS	MS	F	*P* value
Cooking loss (%)	5	959.568	191.914	80.260	0.000
Error	12	28.694	2.391		
Total	17	668.370			

Supplementary Materials S2. Calculated errors of the chemical composition

Table S24 Standard errors of protein

Fish types	TL	BJ	MM	PM	IE	AC

Standard error	0.029	0.144	0.029	0.029	0.115	0.115

Table S25 Standard errors of fat

Fish types	TL	BJ	MM	PM	IE	AC
Standard error	0.087	0.006	0.012	0.000	0.046	0.006

Table S26 Standard errors of ash

Fish types	TL	BJ	MM	PM	IE	AC
Standard error	0.030	0.004	0.031	0.038	0.026	0.008

Table S27 Standard errors of TVB-N

Fish types	TL	BJ	MM	PM	IE	AC
Standard error	0.404	0.140	0.162	0.751	0.440	0.237

Table S28 Standard errors of total protease activity

Fish types	TL	BJ	MM	PM	IE	AC
Standard error	0.286	0.215	0.877	0.662	0.621	0.529

Table S29 Standard errors of cathepsin B

Fish types	TL	BJ	MM	PM	IE	AC
Standard error	0.412	0.103	0.206	0.619	0.309	1.134

Table S30 Standard errors of cathepsin L

Fish types	TL	BJ	MM	PM	IE	AC
Standard error	1.083	0.120	0.120	0.241	0.601	0.722

Table S31 Standard errors of cathepsin D

Fish types	TL	BJ	MM	PM	IE	AC
Standard error	0.152	0.000	0.152	0.152	0.760	0.456

Table S32 Standard errors of calpain

Fish types	TL	BJ	MM	PM	IE	AC
Standard error	0.385	0.289	0.000	0.096	0.289	0.192

Table S33 Standard errors of serine protease

Fish types	TL	BJ	MM	PM	IE	AC
Standard error	0.933	0.833	0.667	0.611	1.313	1.162
